# Dietary Patterns and Nutrient Intake in Pregnancy and Lactation

**DOI:** 10.3390/nu17091543

**Published:** 2025-04-30

**Authors:** Gail Rees, Louise Brough

**Affiliations:** 1 School of Biomedical Sciences, University of Plymouth, Drake Circus, Plymouth, Devon PL4 8AA, UK; 2 School of Food Technology and Natural Sciences, College of Sciences, Massey University, Palmerston North 4410, New Zealand; l.brough@massey.ac.nz

Optimal nutrition during pregnancy and lactation is essential for the health and well-being of both the mother and the developing child. Fetal physiological development and metabolism are significantly influenced by maternal diet, which can determine the future health and disease risk of the offspring [1]. Furthermore, a mother’s nutritional status during pregnancy affects her breast milk composition, her health in subsequent pregnancies, and the global burden of chronic diseases [2].

Determining optimal nutrition for different stages of pregnancy and lactation presents unique challenges. The following Special Issue brings together global research that explores diverse dietary patterns, adherence to dietary guidelines, and associations between dietary patterns and health outcomes. By focusing on dietary patterns rather than individual nutrients, we gain a more comprehensive understanding of how diet influences maternal and fetal/infant health. Nevertheless, there are several individual nutrients that are often lacking in people’s diets worldwide and pose a particular challenge during pregnancy and lactation. In this Special Issue, we provide an in-depth exploration of the impact of vitamin D status and iron intake during pregnancy and lactation.

In a study examining 76 breastfeeding women, Jin et al. (2025) (Contribution 1) present food and nutrient intakes from 4-day weighed intake diaries and compare their findings to dietary guidelines. None of the women included in the study met the current New Zealand recommendations for all four food groups (fruits, vegetables, grain foods, meats, and milk/milk products), and many of them were noted as having nutrient intakes below the estimated average requirement. The women were noted as having particularly low intakes of vitamins E and D, manganese, and selenium. These findings align with those of a previous study involving pregnant women in New Zealand, in which it was found that only 3% of participants met all food group recommendations [3]. Further research is required to understand the barriers to healthy eating, and targeted initiatives are needed to enable healthy behaviours and ensure optimal health for both mothers and their infants.

Maintaining an adequate vitamin D status remains a challenge across the world. Vitamin D deficiency causes osteomalacia in adults, and a low status during pregnancy and lactation can contribute to the occurrence of rickets in offspring [4]. Low vitamin D status is common in Northern European countries, such as the UK, where there is insufficient sunlight over the winter months [5]. Deficiency is particularly common in women who cover their skin for modesty or religious reasons, and deficiency has been reported in Iran [6] and Saudi Arabia [7], in addition to countries where individuals must take measures to protect themselves from the strong sunlight, such as Australia [8]. In this Special Issue, Meija et al. (Contribution 2) report on vitamin D intake and serum concentrations in pregnant and post-partum women in Latvia. The median serum vitamin D concentration was found to be 34.0 ng/mL, and only a minority of pregnant women (21.9%) were found to have a serum vitamin D concentration >45 ng/mL. The authors found no association between dietary intake and serum concentrations of vitamin D; however, they did find a significant correlation with the use of vitamin D supplements (r = 0.41; *p* < 0.001 in pregnant women and r = 0.35; *p* < 0.001 in postpartum women). The results of this study highlight the need for year-round vitamin D supplementation during pregnancy and the post-partum period for optimal serum concentrations in pregnant women.

Plant-based diets are growing in popularity as individuals are encouraged to adopt more sustainable food habits [9]. While plant-based diets can theoretically cover all nutrient needs for most individuals, care must be taken to ensure adequate intake during pregnancy and breastfeeding. The results of a survey of over 1000 women in Poland conducted by Przybysz et al. (Contribution 3) showed that consuming a plant-based diet 6 months before pregnancy and during pregnancy did not change the incidence of GDM, anaemia, and gestational hypertension. Furthermore, no association was found between diet type before conception and delivery method or newborn birth weight. The study authors concluded that those following a plant-based diet during the preconception period had similar obstetric outcomes to those following an omnivore diet.

Across the world and within communities, different dietary patterns have been identified and consistently linked to health outcomes. Different scoring methods exist to characterise diets, such as the Dietary Inflammatory Index [10]. One paper included in our Special Issue by Zhi et al. (Contribution 4) explores the association between the Dietary Inflammatory Index and hyperemesis gravidarum (HG). The incidence rate of HG ranges from 0.3% to 10.8% [11], and the condition is characterised by severe and persistent nausea and vomiting during pregnancy, which can cause dehydration, electrolyte disorders, weight loss, and ketosis, often necessitating hospitalisation [12]. More than 2000 pregnant women in China completed a semi-quantitative food frequency questionnaire, and their Dietary Inflammatory Index (DII) scores were examined. After adjusting for confounders, individuals with the highest tertile of DII score were found to have a higher risk of HG (OR = 1.65, 95% CI: 1.04, 2.62, P trend = 0.032). This association was stronger in those who were overweight/obese during the pre-pregnancy period (P interaction = 0.018). This finding suggests that dietary recommendations for HG should focus on minimising the DII through the incorporation of foods rich in anti-inflammatory components such as monounsaturated fatty acids, polyunsaturated fatty acids, fiber, vitamin B6, folic acid, niacin, riboflavin, thiamin, vitamin A, vitamin C, vitamin D, vitamin E, zinc, selenium, and magnesium.

The final paper included in this Special Issue explores the impact of prenatal dietary patterns on child neurodevelopment (Ouyang et al.) (Contribution 5). As with previous studies, the authors adopted an approach whereby dietary patterns were used to explore the impact of diet during pregnancy and outcomes in offspring. Neurodevelopment was assessed at 36 months, and the mothers’ diet was assessed over three trimesters using food frequency questionnaires. Five maternal dietary patterns were identified through principal component analysis, namely, protein- and micronutrient-rich dietary pattern; low-iron dietary pattern; pasta as the staple food; iron-rich dietary pattern; and tuber, fruit, and baked food dietary patterns. The findings showed that children of mothers who followed a high-protein and micronutrient-rich dietary pattern had higher gross motor and problem-solving scores. Children of women who followed a low-iron dietary patterns during the first trimester had lower problem-solving scores, with those exposed to a low-iron dietary pattern in the second and third trimesters having lower gross motor scores. Furthermore, the children of mothers who followed a low-iron dietary pattern in the third trimester had lower communication scores. A balanced protein- and micronutrient-rich dietary pattern and adequate iron dietary intake throughout pregnancy may be beneficial to children’s neurodevelopment.

As we continue to unravel the complexities of nutrition during pregnancy and lactation, it is imperative to adopt a holistic approach that considers cultural, socioeconomic, and individual variations in dietary patterns. This Special Issue aims to foster further research and collaboration in this vital field, ultimately contributing to improved health outcomes for mothers and their children worldwide.

We extend our gratitude to all of the authors, reviewers, and contributors who have made this Special Issue possible. We hope that the insights gained from these studies will inspire continued advancements in maternal and infant nutrition research.
